# Comparative assessment of radiation therapy–induced vasculitis using [^18^F]FDG-PET/CT in patients with non-small cell lung cancer treated with proton versus photon radiotherapy

**DOI:** 10.1007/s00259-023-06535-3

**Published:** 2023-12-14

**Authors:** D. Evanson, M. Griffin, S. E. O’Reilly, T. Johnson, T. Werner, E. Kothekar, P. Jahangiri, C. B. Simone, S. Swisher-McClure, S. J. Feigenberg, M.-E. Revheim, J. Zou, A. Alavi

**Affiliations:** 1 Drexel University College of Medicine, Philadelphia, PA USA; 2 Department of Radiology, University of Pennsylvania, Philadelphia, PA USA; 3 Department of Radiation Oncology, University of Pennsylvania, Philadelphia, PA USA; 4 University of Notre Dame, Notre Dame, IN USA; 5 New York Proton Center, New York, NY USA; 6 The Intervention Center, Oslo University Hospital, Oslo, Norway; 7 Institute of Clinical Medicine, Faculty of Medicine, University of Oslo, Oslo, Norway

**Keywords:** Radiation therapy, Vasculitis, Proton radiotherapy, Photon radiotherapy, [^18^F]FDG-PET

## Abstract

**Purpose:**

To assess radiation therapy (RT)-induced vasculitis in patients with non-small cell lung cancer (NSCLC) by examining changes in the uptake of ^18^F-fluoro-D-deoxyglucose ([^18^F]FDG) by positron emission tomography/computed tomography (PET/CT) images of the ascending aorta (AA), descending aorta (DA), and aortic arch (AoA) before and after proton and photon RT.

**Method:**

Thirty-five consecutive locally advanced NSCLC patients were definitively treated with proton (*n* = 27) or photon (*n* = 8) RT and concurrent chemotherapy. The patients were prospectively enrolled to undergo [^18^F]FDG-PET/CT imaging before and 3 months after RT. An adaptive contrast-oriented thresholding algorithm was applied to generate mean standardized uptake values (SUVmean) for regions of interest (ROIs) 3 mm outside and 3 mm inside the outer perimeter of the AA, DA, and AoA. These ROIs were employed to exclusively select the aortic wall and remove the influence of blood pool activity. SUVmeans before and after RT were compared using two-tailed paired *t*-tests.

**Results:**

RT treatments were associated with increased SUVmeans in the AA, DA, and AoA—1.9%, 0.3%, and 1.3% for proton and 15.8%, 9.5%, and 15.5% for photon, respectively. There was a statistically significant difference in the ∆SUVmean (post-RT SUVmean − pre-RT SUVmean) in patients treated with photon RT when compared to ∆SUVmean in patients treated with proton RT in the AA (*p* = 0.043) and AoA (*p* = 0.015). There was an average increase in SUVmean that was related to dose for photon patients (across structures), but that was not seen for proton patients, although the increase was not statistically significant.

**Conclusion:**

Our results suggest that patients treated with photon RT for NSCLC may exhibit significantly more RT-induced inflammation (measured as ∆SUVmean) in the AA and AoA when compared to patients who received proton RT. Knowledge gained from further analyses in larger cohorts could aid in treatment planning and help prevent the significant morbidity and mortality associated with RT-induced vascular complications.

**Trial registration:**

NCT02135679.

## Background

Lung cancer is the leading cause of cancer-related deaths worldwide. Lung cancer causes nearly one in five (18.0%) cancer-related deaths and accounts for over one in ten (11.4%) of all cancer diagnoses [1]. In 2020, there were 1.8 million deaths that occurred because of lung cancer and 2.2 million new lung cancer diagnoses [1]. Of all lung cancers, non-small cell lung cancer (NSCLC) accounts for over 85% of cases [2, 3]. Radiation therapy (RT) is a standard treatment, frequently employed in locally advanced NSCLC as either definitive, neoadjuvant, or adjuvant treatment [4–6].

Despite recent advancements in RT in NSCLC, such as intensity-modulated radiation therapy (IMRT), image-guided radiotherapy, four-dimensional computed tomography treatment planning, and various motion mitigation strategies, RT toxicity is an increasingly recognized cause of morbidity and mortality [7–9]. RT has been associated with a significant risk for treatment-induced toxicities, including radiation pneumonitis, radiation-induced esophagitis, radiation-induced heart disease, and radiation-induced vasculitis [4, 10, 11]. Still, consensus on the optimal approach to screen for and manage these toxicities is limited [12]. Without clear data on the risks of toxicities, clinicians must balance optimizing local disease control with the risk of radiation-induced sequelae and consider delivering sub-optimal RT targeting to avoid excessive irradiation dose to sensitive centrally located mediastinal structures [13].

In RT-induced vasculitis, there is a narrowing of blood vessels in areas close to the field of radiation. The narrowing of blood vessels occurs from the upregulation of a specific transcription factor, nuclear factor–kappa B, due to chronic oxidative stress from RT [14]. Patients typically present with fever, weight loss, malaise, and arthralgia that may lead to complications, including loss of vision, renal failure, myocardial infarction, and vessel necrosis [15]. The decreased blood flow from vasoconstriction and inflamed vascular walls may ultimately result in vascular dementia, brain damage, and death [16]. Patients with head and neck cancer who received RT have been reported to be at an elevated risk of ischemic stroke (relative risk 2.16), and cerebrovascular mortality occurred in 13.3% of patients within 10 years of receiving RT-only treatment [17, 18]. Due to the heterogeneity of cases of vasculitis, each patient requires an individualized approach to their treatment [13]; first-line management of vasculitis often includes a short course of corticosteroids [15].

**Fig. 1:**
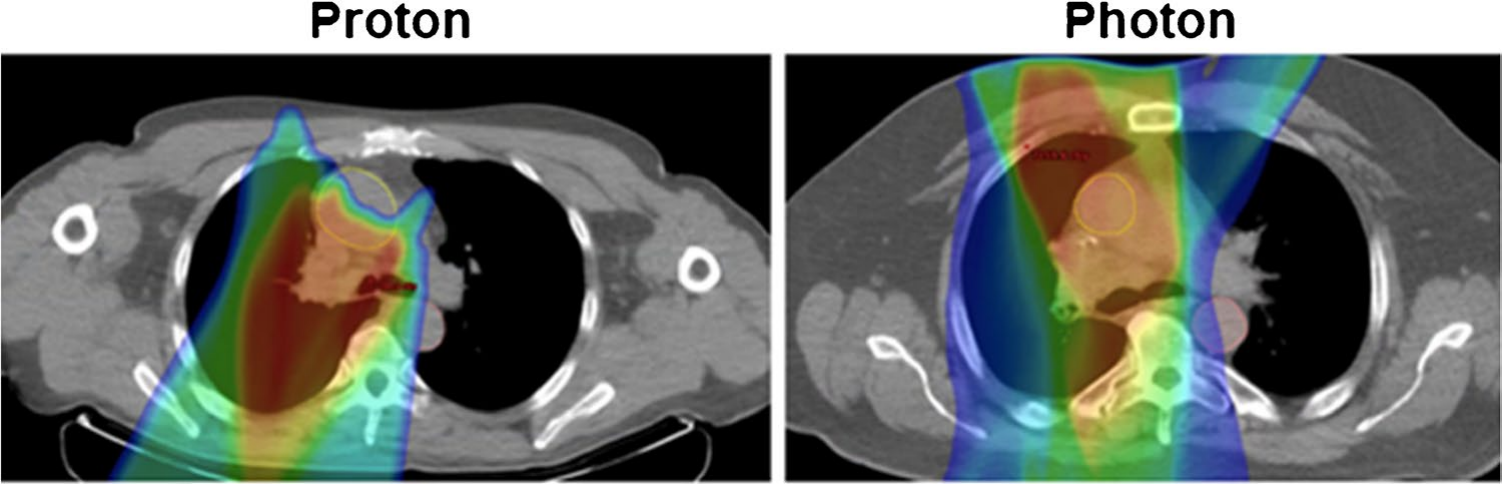
Representative proton and photon patient dose distribution comparison

Photon-based RT is the standard treatment of choice for patients with NSCLC. However, multiple studies have shown that the use of proton RT may improve outcomes, with a potential to decrease inflammation by up to 66% and reduce the risk of pneumonitis [12, 19–23]. Proton therapy has been recognized as an option to better spare critical surrounding tissues from excessive radiation due to the Bragg Peak distribution pattern [24]. It is a promising modality that may improve outcomes without increasing and even reducing side effects in lung cancer patients due to the lack of exit dose and conformal dose distributions achieved (Fig. 1) [22]. To date, cost and access have limited the use of proton RT [19]. Further, there is limited knowledge on intramodality in the practice of proton-based therapy due to the anatomical variations among patients [21]. Therefore, it is very timely to conduct studies that focus on not only the effect of radiotherapy on the direct organ or tissue but also on the therapy’s toxic side effects on surrounding organs and tissues. Most recently, a study that utilized ^18^F-fluoro-D-deoxyglucose ([^18^F]FDG) positron emission tomography/computed tomography (PET/CT) to quantify RT-induced inflammation following proton and photon therapy in the lungs concluded that there was a lower degree of inflammation in the lungs with proton therapy [19]. Our study was designed to compare proton and photon RT in terms of RT-induced inflammation.

While the utility of [^18^F]FDG-PET in the quantitative assessment of inflammation has been validated over the past three decades in many settings, our group is the first to apply this technique in the assessment and direct comparison of proton and photon RT-induced vascular inflammation [4, 23, 25–27]. In this prospective research study, we compared proton and photon RT-induced inflammation by examining changes in the uptake of [^18^F]FDG before and after RT for the ascending aorta (AA), descending aorta (DA), and aortic arch (AoA) in 35 patients with locally advanced NSCLC.

## Methods and materials

### Study population

Upon receipt of Institutional Review Board approval for prospective data collection and image analysis with Health Insurance Portability and Accountability Act waiver, this prospective study was performed at the Hospital of the University of Pennsylvania. This study assessed a total of 35 consecutive patients with locally advanced NSCLC treated with definitive concurrent chemotherapy and radiation therapy. Patients were separated into proton and photon RT subgroups, with 27 receiving proton RT and 8 receiving photon RT (Table 1). Of the 27 treated with proton RT, 17 received carboplatin, 3 received cisplatin, 8 received etoposide, 14 received paclitaxel, and 10 received another form of chemotherapy. Of the 8 treated with photon RT, 5 received carboplatin, 2 received cisplatin, 3 received etoposide, 5 received paclitaxel, and 1 received another form of chemotherapy. All patients prospectively enrolled in this trial underwent [^18^F]FDG-PET/CT imaging before and approximately 3 months after RT. Patients received prescription doses of 60.0–66.6 Gy in 1.8–2.0 Gy daily fractions. All patients were imaged on the same PET/CT scanner.

**Table 1:** Patient characteristics

Characteristic	Proton RT	Photon RT
No. of patients	27	8
Male	12	4
Female	15	4
Age in years (mean/median)	70/68	60/60
Stage		
IB	1	0
IIA	1	0
IIB	1	0
IIIA	19	3
IIIB	5	5
Chemotherapy		
Carboplatin	17	5
Cisplatin	3	2
Etoposide	8	3
Paclitaxel	14	5
Other	10	1
Planning target volume		
Volume in cm mean (range)	458.1 (240.7–1690.6)	482.0 (125.5–1154.5)
Equivalent Sphere Diameter in cm mean (range)	9.3 (7.7–14.8)	9.3 (6.2–13.0)

### [^18^F]FDG PET/CT image acquisition and scans

Patients fasted for at least 6 h prior to receiving 555 MBq (15 mCi) of [^18^F]FDG administered intravenously approximately 60 min before image acquisition. The serum glucose levels of patients enrolled were less than 200 mg/dL before injection of [^18^F]FDG. Scans were acquired utilizing a 16-detector row LYSO PET/CT scanner with time-of-flight data acquisition (Gemini TF; Philips Healthcare, Bothell, WA). [^18^F]FDG PET/CT images were obtained from the base of the skull to the mid-thigh approximately 60 min after [^18^F]FDG injection with 3 min bed positions. Image reconstruction was performed (using a list-mode maximum-likelihood expectation-maximizing algorithm) with 33 ordered subsets and 3 iterations. Energy-rescaled low-dose CT images were used for attenuation correction of PET images. Slice thickness was utilized on PET and CT acquisition to allow for fusion. PET images were corrected for attenuation, random coincidences, and scatter correction.

The average interval between the completion of RT and post-treatment [^18^F]FDG-PET/CT was 101 days (range of 75–179 days). A total of 105 aorta PET measurements (one for each ROI in the AA, DA, and AOA) were evaluated pre- and post-RT for this study. Table 1 provides an overview of the characteristics of the patient cohort, and Table 2 provides the different RTs administered to each patient and the average doses administered. A common preexisting diagnosis among the study cohort was chronic obstructive pulmonary disease (COPD); however, no other preexisting vascular pathology was reported. No inflammatory conditions were identified.

**Table 2:** Comparison of irradiation doses received by cardiac structures for proton (P) and photon (X) patients

Vascular structure	Proton mean (cGy)	Photon mean (cGy)	Mean difference (X − P) (cGy)	Proton max (cGy)	Photon max (cGy)	Max difference (X − P) (cGy)
Ascending aorta	3089	4874	1785	6723	6861	138
Descending aorta	1737	1987	250	6018	5289	− 729
Arch of the aorta	3699	3133	− 682	6232	6001	− 231

### Quantification analysis and statistical analysis

**Fig. 2:**
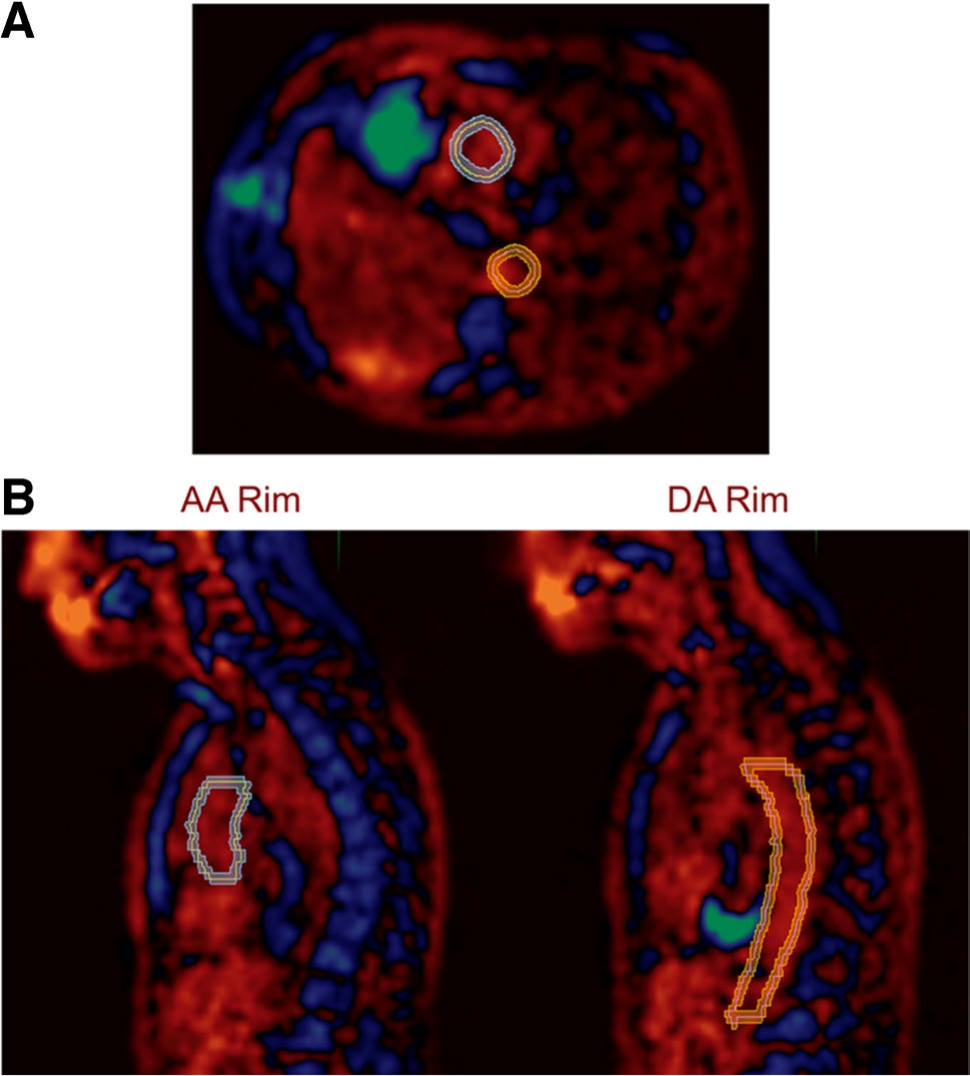
Illustration demonstrating how regions of interest (ROI) were created to encompass the region 3 mm outside and 3 mm inside the outer perimeter in the ascending aorta (AA) and descending aorta (DA), in transaxial (**A**) and sagittal views (**B**)

The AA, DA, and AoA were contoured, and a region of interest (ROI) was created that encompassed the region 3 mm outside and 3 mm inside the outer perimeter of each vascular structure to represent the structure wall and remove the influence of blood pool activity (Fig. 2). The mean standardized uptake values (SUVmean) were generated for the ROIs that corresponded to each vascular structure. SUVmeans before and after RT were compared using two-tailed paired *t*-tests. Differences were considered statistically significant when the two-tailed *p*-value was less than 0.05.

## Results

For patients who received proton RT, 12 were males and 15 were females, with a mean age of 70 years. Proton RT patients had predominately stage IIIA (70.4%) or IIIB (18.5%) disease. For patients who received photon RT, four were male and four were female, with a mean age of 60 years. Photon therapy patients had exclusively stage IIIA (62.5%) or IIIB (37.5%) NSCLC. The patients in both cohorts received various standard concurrent platinum-based doublet chemotherapy, most commonly with carboplatin plus paclitaxel or with cisplatin plus etoposide. The mean doses of proton/photon RT to the AA, AoA, and DA were 30.9/49.5 Gy, 37.0/30.2 Gy, and 17.4/20.6 Gy, respectively. Patient and baseline characteristics are summarized in Table 1.

**Fig. 3:**
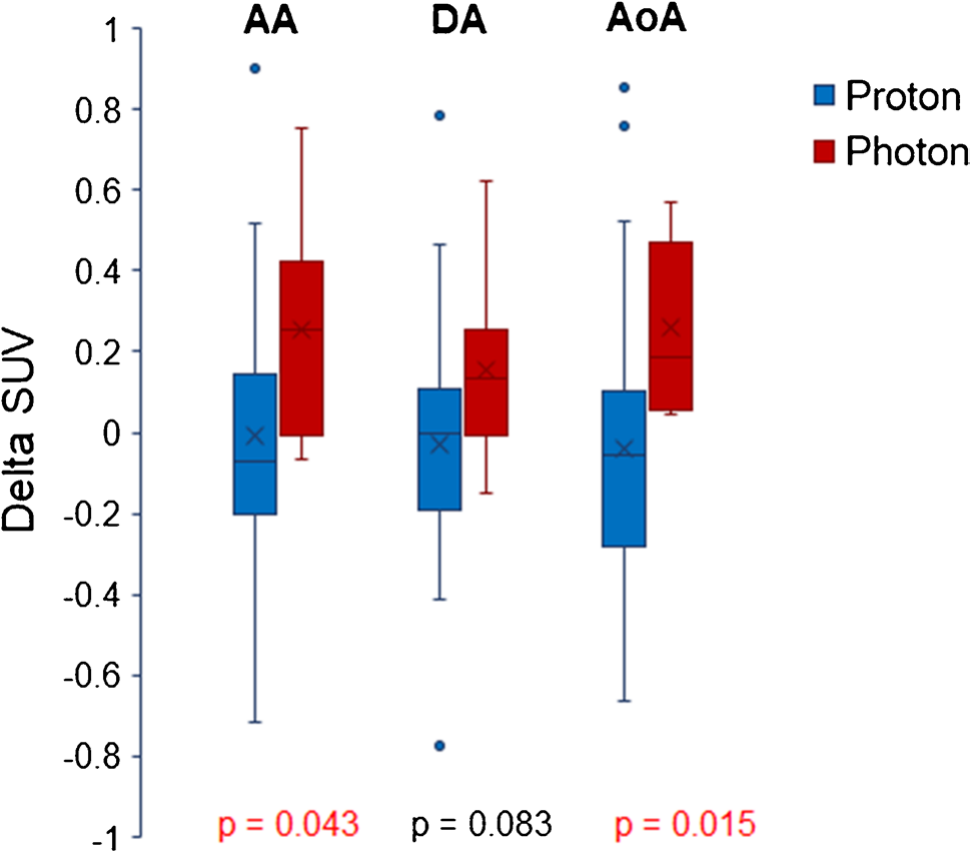
∆SUVmean for ascending aorta (AA), descending aorta (DA), and aortic arch (AoA) for patients treated with proton or photon RT

There was an increase in SUVmean for the AA, DA, and AoA that was much more evident with proton RT (1.9%, 0.3%, and 1.3% for proton RT; 15.8%, 9.5%, and 15.5% for photon RT, respectively), with the increase being statistically significant (*p* < 0.05) for AA and AoA in patients receiving photon RT compared to proton RT. The ∆SUVmeans in the photon cohort were significantly different from those in the proton cohort in the AA (*p* = 0.043) and the AoA (*p* = 0.015) (Fig. 3).

**Fig. 4:**
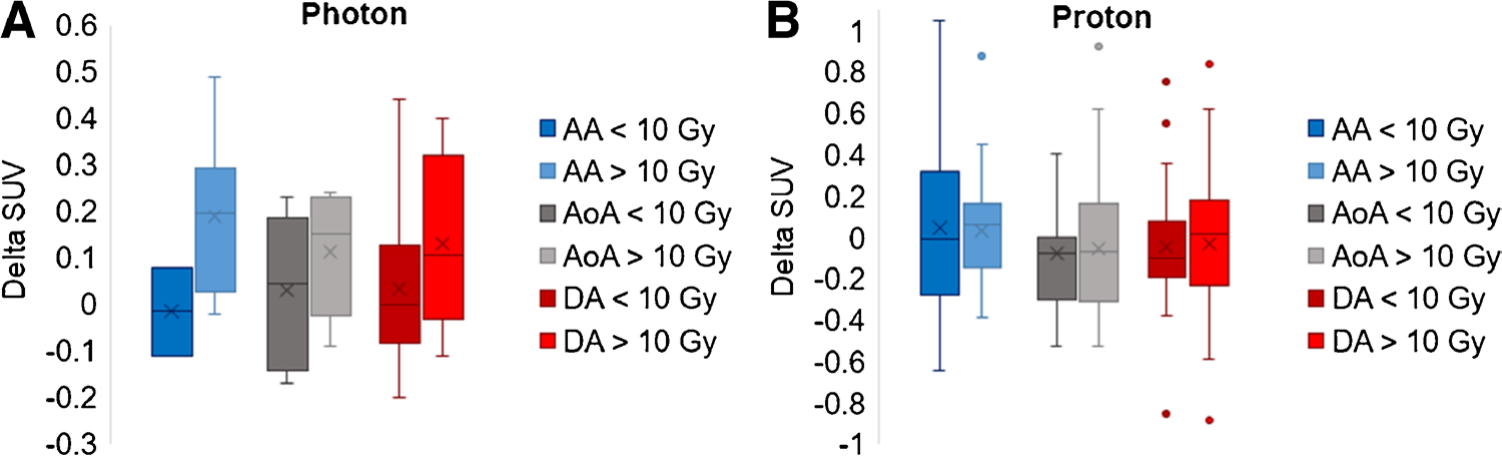
Comparison of rim structures (AA, AoA, and DA) and dose received (portion of structure receiving greater than or less than 10 Gy) for **A** photon patients and **B** proton patients

When examining the rim structures and dose received (portion of structure receiving greater than or less than 10 Gy), there was an increase in mean SUVmean related to dose for the photon patients (average increase across structures of 0.13SUV), which was not evident in the proton patients (0.01SUV); however, these differences were not statistically significant (Fig. 4).

To add perspective on the relative irradiation field sizes between the photon and proton populations in this study, the equivalent sphere diameter of the Planning Target Volume (PTV) and PTV volume is reported in Table 1. The equivalent sphere diameter mean is the same (9.3 cm) between the two patient populations (*p* = 0.98) and the difference between the PTV volumes is also not statistically significant (*p* = 0.85). While the average PTV volume is slightly smaller for the proton cohort, the minimum and maximum volumes are higher (as observed in the range). To illustrate the target location relative to the cardiac structures, we can look at the maximum doses of structures. When comparing the patient cohorts’ maximum doses to the AA, DA, and AoA using a 2-sided Student’s *t*-test, the results were not statistically significant (*p* = 0.69, *p* = 0.47, *p* = 0.77, respectively).

## Discussion

We observed an increased [^18^F]FDG uptake in the AA, DA, and AoA after both proton and photon RT, with significantly greater uptake in the AA and AoA of patients treated with photon RT. The uptake in DA was not significantly different but yielded a 9.2% additional increase in SUVmean with photon RT compared to proton RT. The non-significance may have been due to the DA being a larger lengthwise structure with inflammation decreased distal to the lungs. The extent of vascular inflammation in the aorta is concerning with both proton and photon RT; however, our results suggest that this effect and subsequent risks of radiation-induced vascular toxicities may be lower with proton RT.

SUVmean increased with dosage in patients treated with photon RT, although this pattern was not evident in patients who received proton RT. This trend was demonstrated in other studies, including Niska et al. who found mean heart RT dose > 15 Gy substantially increased the risk of vasculitis [28]. Twitchell et al. found that a lack of clinical evidence and guidelines in head and neck cancers led to individuals who underwent RT-developed delayed vasculitis [13]. This dose-dependent effect demonstrates the importance of early recognition of vasculitis in patients who undergo multiple RT treatments. Long-term survival of patients with vasculitis highly depends on the diagnosis, response to therapy, and adverse effects of immunosuppressive drugs for treating infections [29]. Additional studies with longer follow-up are warranted to examine the effects of the RT treatment modality on all-cause mortality.

Because of the small cohort (*n* = 35) with a short time follow-up frame (3 months), the power of our study is limited. Additional limitations include the lack of random treatment assignment, variability in the time between imaging and RT, and the potential confounding effects of comorbid cardiovascular disease in our patient population. Patients with larger, more difficult-to-treat tumors were more likely to receive proton RT since this treatment was more likely to be approved by payors for patients with more advanced disease. If this bias was evident, it would have worked against the difference we observed because patients treated with proton RT may have required larger irradiation fields, leading to increased risks of inflammation in the nearby vasculature. Similarly, patients in the proton RT cohort were older than their photon counterparts, likely due to differential insurance approval patterns, which could have served to further select patients with preexisting cardiac and vascular subclinical morbidity to preferentially receive proton therapy. As of result of these potential biases, the observed difference in RT-induced inflammation may actually be an underestimate of the true difference between proton RT and photon RT. The location of the tumor and the extent of the RT field were not taken into consideration, which could have affected the aortic wall to a varying degree based on proximity to the tumor volume. However, previous research has shown that proton therapy is used for larger tumors that would require larger RT-fields [20]. Therefore, this bias would have increased inflammation seen in proton RT patients, whereas actual inflammation was significantly lower with proton RT.

Additionally, there are limitations that need to be considered when interpreting the results of the present study since the data collected were based on their clinical relevance. There was a varying time between imaging and RT and between RT and follow-up. The latter may have led to detecting lower degree of vascular inflammation due to the longer interval between treatment and PET imaging. However, the interval between proton and photon RT was not significantly different. Part of this variance was important to remove any influence of differences in chemotherapy agents received. We ensured that all patients had a scan prior to starting chemotherapy and RT and were not given chemotherapy and RT the last 4–6 weeks before the follow-up [^18^F]FDG PET/CT scan. Finally, the [^18^F]FDG PET/CT performed followed a standard clinical protocol for the population examined and was not optimized for the imaging of vascular inflammation for research purposes. Further, recent investigations recommend that imaging be delayed 90 to 180 min to assess specifically inflammation in the vascular wall [30, 31]. A delayed acquisition period has been shown to increase [^18^F]FDG uptake in the vascular wall and decrease blood pool activity, which therefore improves the contrast resolution of this technique [32]. Our study utilized clinical data and therefore did not follow these recommendations which could have impacted the results we generated. The patient cohort was originally enrolled for detecting circulating tumor cells [33, 34]. Therefore, limited spatial resolution causing intensity diffusion (partial volume effect) hampers the correct delineation of small or thin structures. Despite our strict efforts in centering of rings on the outer and inner aortic wall, correction for the partial volume effect was extremely challenging. The aortic wall is very thin (no more than 1–2 mm), and therefore, such corrections will be fraught with errors and poor reproducibility. Furthermore, the images were acquired at 60 min, and as such, blood pool activity was substantial, which made partial volume correction more complicated. We have used this approach in our research in the past but could not justify its relevance for this project. We believe delayed imaging at 2–3 h and adopting high-resolution total body PET instruments will eliminate the difficulties that are faced with current methodologies in the future. In spite of these shortcomings, the data generated in this study supported our hypothesis and provided relatively accurate data for pursuing such research in the future.

## Conclusions

Our results suggest that patients treated with photon RT may exhibit significantly more radiation-induced inflammation as revealed by [^18^F]FDG PET in the AA and AoA, compared to patients who received proton RT. However, further research into this area is necessary for future prospective research studies. Knowledge gained from further analyses in larger cohorts may aid in treatment planning decisions and help reduce the morbidity and mortality associated with RT-induced vasculitis. Future research comparing the RT-induced inflammation of aortic structures in patients with both upper and lower lung lobe lesions may be especially fruitful. Additionally, the segmentation of other large vessels that are not included in the irradiation field could provide important findings in this population. A thorough comparison of current RT modalities (photon and proton therapies) with a larger cohort of individuals and longer follow-up is necessary to evaluate the validity of our results and inform future RT treatment decisions.

## Declarations

### Availability of data and materials

The datasets used and/or analyzed during the current study are available from the corresponding author on reasonable request.

## Abbreviations

RT: Radiation therapy
NSCLC: Non-small cell lung cancer
[^18^F]FDG: ^18^F-fluoro-D-deoxyglucose
PET/CT: Positron emission tomography/computed tomography
AA: Ascending aorta
DA: Descending aorta
AoA: Aortic arch
SUVmean: Mean standardized uptake values
ROIs: Regions of interest
PTV: Planning target volume
∆SUVmean: Post-RT SUVmean − pre-RT SUVmean
IMRT: Intensity-modulated radiation therapy
COPD: Chronic obstructive pulmonary disease
CCRT: Concurrent chemoradiotherapy
